# Supplementary figures and images for: Small change, big difference: A promising praziquantel derivative designated P96 with broad-spectrum antischistosomal activity for chemotherapy of schistosomiasis japonica
Source: PLoS Negl Trop Dis. 2023 Jul 6;17(7):e0011215. doi: 10.1371/journal.pntd.0011215 (PMC10353801; doi:10.1371/journal.pntd.0011215)

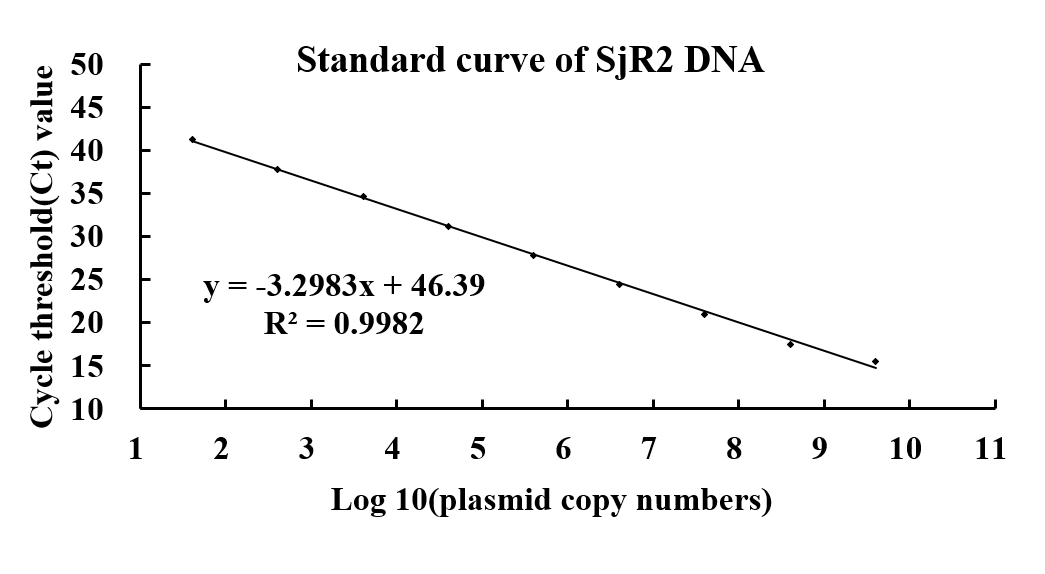

Supplement: S1 Fig — The target DNA sequence of SjR2 was cloned into plasmids using pMD20-T II cloning reagent Kit (Tiangen, Beijing, China), and the plasmids were purified by TIAN pure Mini Plasmid Kits (Tiangen, Beijing, China). Sequencing of the cloned amplification product confirmed that it was identical to part of the Schistosoma japonicum retrotransposon SjR2. The plasmid was tested in a series of 10-fold dilutions by quantitative real-time quantitative PCR. A standard curve of SjR2 DNA was constructed, resulting in a detection limit of 4.0 copies of SjR2 DNA. The correlation coefficient (R2) was 0.9982 and the slope was -3.2983. (TIF) [file pntd.0011215.s001.tif]
